# Physical Health Mediates the Relationship between Psychological Well-Being and Engagement in Exercise across Age in a German Sample

**DOI:** 10.3390/jfmk3030039

**Published:** 2018-07-03

**Authors:** Jillian Minahan, Karen L. Siedlecki

**Affiliations:** Department of Psychology, Fordham University, Bronx, NY 10458, USA

**Keywords:** older adults 1, aging 2, exercise 3, health 4, well-being 5

## Abstract

The prevalence of chronic illness among middle-aged and older adults is increasing worldwide as the population continues to age. One way to prevent the continued increase and subsequent negative outcomes of chronic illness is to increase the number of individuals who engage in exercise. Thus, it is important to examine which factors predict engagement in exercise in middle-aged and older adults. As a result, the current study examined the relationship between physical health, psychological well-being, and engagement in exercise in a sample of middle-aged and older German adults. We found that increased age was associated with less frequent engagement in exercise. We also found that physical health mediated the relationship between psychological well-being and engagement in exercise. Finally, we found that age did not moderate the relationship between subjective well-being and engagement in exercise, suggesting that the role of physical health as a mediator was similar in older adults compared to middle-aged adults. These findings have important implications for interventions seeking to promote exercise among adults.

## 1. Introduction

The average life expectancy in Europe and the proportion of older adults (65 years and older) in the population has been increasing since the twentieth century. For example, in 2010, individuals who were 65 years and older comprised 17.4% of the total European population, and this proportion is projected to increase to 29.5% by 2060 [1]. Germany has one of the highest proportions of individuals aged 65 and older in Europe and must consider the unique health needs of their large older adult population [2]. One complication of increased age is the increased likelihood of experiencing chronic illness. Complications related to cardiovascular disease, cancer, and other chronic conditions and noncommunicable diseases are among the top 10 leading causes of death in Europe [2]. The rates of chronic illness worldwide, and in Germany specifically, are significant, and their negative impact is likely to be substantial as the population continues to age.

For instance, previous research indicated that in Germany, approximately 50% of all adults aged 65 and older had been diagnosed with at least three chronic illnesses [3,4]. The increased prevalence may have significant financial implications as well. For instance, a recent review found that the increase in individuals being diagnosed with more than one chronic illness has been linked to increases in doctor visits, hospitalizations and use of medication, as well as the associated healthcare costs, such as medication and out-of-pocket costs [5]. Expenditures for chronic illness healthcare are likely to continue to increase as the population ages and becomes increasingly afflicted with chronic illnesses, potentially straining the healthcare system. Additionally, this increase of chronic illness and disease comorbidity will likely be related to poor quality of life for many more individuals as poor health status has been found to be related to negative psychological outcomes, such as depression e.g., [6], negative affect e.g., [7], and low quality of life e.g., [8]. Clearly, the negative implications of the rise of chronic illness are substantial.

### 1.1. Prevention of Chronic Illness through Health-Promotive Behaviors

Although this increase in the prevalence of health conditions is likely to continue to have negative consequences, previous research has examined ways in which they can be prevented or reduced. For instance, the World Health Organization (WHO) reports that engaging in exercise can reduce one’s risk of or help to manage many of these chronic health conditions, including cardiovascular disease and dementia [5,9]. Koeneman, Verheijden, Chinapaw and Hopman-Rock defined exercise as “physical activity, which is planned, structured, and repetitive, with the specific goal to maintain or improve physical fitness” [10] (p. 2) while they defined physical activity as “unstructured activities incorporated in daily life” [10] (p. 1). Throughout this study, we focused on the more structured and intentional activity of “exercise” rather than the more general “physical activity”.

Engaging in exercise has also been found to be related to psychological well-being e.g., [11]. Likewise, some risk factors of noncommunicable diseases, such as obesity and hypertension, can be managed through engagement in exercise. To experience the health benefits of exercise, the WHO recommends that adults should engage in at least 150 min of moderate-intensity or 75 min of vigorous-intensity physical activity per week [9].

Despite the advantages that engaging in exercise provides, especially to older adults, many individuals do not follow the recommendations provided by the WHO. For instance, estimates from a WHO fact sheet suggest that 26% of men and 35% of women residing in high-income countries are not physically active to the extent recommended by the WHO [9]. In Germany specifically, a large, nationally representative study found that 72.8% of women and 65.3% of men aged 65 years and older did not meet the WHO recommendations for physical activity. More significantly, 48.2% of women and 52.8% of men over the age of 65 did not engage in any exercise [12]. Furthermore, previous research found that older adults engaged in less physical activity and exercise than younger adults [13]. However, research has found that older adults who begin an exercise regime, whether it is cardiorespiratory endurance or resistance training activities, show improvements in physical capacity [14]. Therefore, one aim of the current study was to examine whether age was a significant predictor of exercise engagement, specifically investigating whether increased age was related to decreased exercise among middle-aged and older adults.

### 1.2. Relations between Physical Health, Psychological Well-Being and Exercise

In addition to differences across age, the present study also examined the influence that two indices of wellness, namely physical health and psychological well-being, had on engagement in exercise. Self-rated health has been found to be related to engagement in physical activity, that included vigorous exercise, such that better adherence to exercise regimens was associated with better health status [15]. Researchers have found that engagement in exercise was also related to physical functioning, namely functional status, in older adults [16]. Additionally, Ku and colleagues found that engagement in exercise was related to the experience of psychological well-being [11]. Taken together, these findings suggest that in addition to physical health indicators, psychological well-being variables should be included in studies to gain a more comprehensive understanding of their relations to engagement in exercise. Thus, the current study examined both physical health and psychological well-being predictors in relation to engagement in exercise.

### 1.3. Determinants of Exercise in Middle-Aged and Older Adults

A majority of the previous literature on the topic examined the impact that exercise has on physical and/or psychological well-being, but few studies have investigated physical health and psychological well-being as determinants of engagement in exercise, especially within a sample of middle-aged and older adults. More specifically, in most of the previous research, engagement in exercise has been conceptualized as the antecedent and the expected benefits, namely improved physical and psychological health, as the outcomes. However, since individuals are under-engaging in exercise, it is important to better understand what factors predict engagement in exercise, so that these factors could serve as possible targets for future intervention to promote exercise.

In a review of the literature regarding interventions to promote physical activity and exercise among older adults, research has enumerated different categories of determinants that have started to emerge in the research as predictors of engagement in exercise. These categories include “personal characteristics, program or regimen-based factors, and environmental factors” [17] (p. 36). The category of personal characteristics includes an individual’s demographic and health as well as attitudinal and psychological factors, and both physical health and psychological well-being may play a role in an older adult’s engagement in exercise [17]. Previous research suggests that poor physical health may be a particularly strong determinant of engagement in exercise compared to other personal characteristics [17]. Therefore, the explanatory influence of physical health in predicting exercise was a focus of the current study. As previous research suggested e.g., [17], although recently determinants of physical activity and exercise among older adults have emerged, there remains a gap in this research as a majority of the literature utilizes only samples of young and middle-aged adults. This suggests the need for more research examining the determinants of exercise among older adults.

Similarly, in a systematic review, researchers found that there was insufficient evidence for most associations between determinants of exercise and engagement in exercise due to studies lacking in rigor and studies examining only one determinant [10]. This conclusion suggests that further research should be conducted to examine the determinants of engagement in exercise. Thus, the current study examined physical health and psychological well-being as determinants of engagement in exercise.

Although few studies have examined the antecedents of exercise in middle-aged and older adults, when antecedents were considered, often only one determinant was included [10]. However, when physical health and psychological well-being were considered together, they were often conceptualized as making direct contributions (i.e., not being mediated by one another) to engagement in exercise [16]. To our knowledge, previous research has not examined a mediation model in which physical health mediated the relationship between psychological well-being and engagement in exercise, and thus, this kind of model was examined in the present study.

In addition, because of the differing levels of physical health status and psychological well-being across age [18,19], the current study examined the moderating effect of age on the relationship between physical health, psychological well-being, and engagement in exercise. Because of age-related health decline, we examined whether the influence of physical health as a mediator between psychological well-being and engagement in exercise varied across age. As previously mentioned, we focused on “exercise” rather than the more general construct of “physical activity.” Although the terms have been used somewhat interchangeably throughout the literature, some researchers have noted a distinction [10]. Thus, we focused our investigation on the more structured and intentional activity of engaging in exercise.

### 1.4. Current Study

The goals of the current study were to (1) examine whether age was negatively associated with engagement in exercise, (2) examine whether physical health (measured by self-rated health, number of illnesses, functional status, and body mass index [BMI]) mediated the relationship between psychological well-being (measured by positive affect, negative affect, life satisfaction, and depressive symptoms) and engagement in exercise, and (3) examine whether age moderated the mediated relationship between physical health, psychological well-being, and engagement in exercise in a sample of middle-aged and older German adults.

Following from the goals of the study, we hypothesized that (1) age would be inversely related to engagement in exercise such that older adults engage in exercise to a lesser extent than younger adults, (2) physical health would mediate the relationship between psychological well-being and engagement in exercise, and (3) age would moderate this mediated relationship given the increase in health decline at advanced ages.

## 2. Materials and Methods

### 2.1. Participants

The data in the present study are from the scientific release of the German Ageing Survey (DEAS), provided by the Research Data Centre of the German Centre of Gerontology (DZA) (https://www.dza.de/en/research/deas.html) [20]. The DEAS is funded by the Federal Ministry for Family Affairs, Senior Citizens, Women and Youth (BMFSFJ). Participant consent was obtained via a joint written request of the DZA and Infas Institute for Applied Social Science. The Fordham University Institutional Review Board approved this investigation utilizing the DEAS data (Approval code: 261; Approval date: 11 November 2015). The DEAS is an ongoing, nationally representative longitudinal survey of community-dwelling German and non-German adults over the age of 40 years. Currently, there are five waves of data collected in 1996, 2002, 2008, 2011 and 2014. Participants were recruited for the DEAS through national probability sampling with stratified sampling by age, gender, and residential location in Germany, namely Eastern or Western Germany. The current study used data from Wave III (collected in 2008), which included 8136 participants, whose ages ranged from 40 to 93 years (*M* = 62.56, *SD* = 11.92) (https://www.dza.de/en/research/deas.html) [20]. The full sample comprised 50.8% males. Wave III was selected for the current study because it comprised the largest number of participants compared to the other waves. The sample was also divided into three age groups for moderation analyses which represented middle-age, young-old and an old-old cohort (i.e., 40–59 years, 60–79 years, 80 years and older). The subsamples comprised 47.6% males, 53.3% males and 51.5% males, respectively.

### 2.2. Measures

#### 2.2.1. Physical Health

Four different observable measures of health were examined to represent the multidimensional latent construct of physical health [8]. These included a global assessment of self-rated health, a subscale of physical functioning (e.g., functional status) from a measure called the Short Form (SF)-36 Health Questionnaire [21,22], number of illnesses, and a measure of BMI. Details of each measure are provided below.

##### Self-Rated Health

Self-rated health was measured by one item that asked “How would you rate your present state of health?” on a Likert scale from 1—“Very good” to 5—“Very bad”. These values were reversed so that higher scores represented better self-rated health. Previous research has found that self-rated health was correlated with a measure of physical functioning (*r* = 0.66, *p* < 0.05) and a measure of emotional health (*r* = 0.65, *p* < 0.05), indicating that it was a valid measure of health with adequate convergent validity [23]. Previous research found similarly good psychometric properties of the single-item self-rated health measure in two longitudinal cohort studies [24], and this item has also been used previously in the DEAS to assess self-rated health [25]. This item was included as an indicator of the latent construct of physical health.

##### Number of Illnesses

The number of illnesses was measured by an 11-item list of illnesses to which participants responded whether they suffer from the illness (1—“Yes”) or not (0—“No”). The listing was adapted from the Charlson Comorbidity Index [26], which has been used to measure disease burden and predict mortality by identifying comorbidities. The illnesses listed within the DEAS included illnesses such as cardiac and circulatory problems, bad circulation, joint, bone, spinal, or back problems, cancer and diabetes. A total sum score was calculated for the number of illnesses each participant reported he/she suffer from at present, which was included as an indicator of the latent construct of physical health.

##### Functional Status

The physical functioning subscale of the SF-36 [21,22] consists of 10 items that measure individuals’ level of functional status by assessing to what extent they are able to carry out activities of daily living (ADLs) on a scale of 1—“Yes, limited a lot”, 2—“Yes, limited a little”, and 3—“No, not limited at all”, meaning higher scores indicate better functioning. Examples of ADLs include carrying groceries, walking one block, and climbing one flight of stairs. Internal consistency of this measure is high (Cronbach’s α = 0.93). The sum of the items was standardized on a 0–100 scale. This summation was substantially negatively skewed. Thus, a logarithmic transformation of functional status subtracted from a constant of 101 (which was the highest possible score of functional limitation plus one) (e.g., LG10 (101-functional status value)) was performed, which improved the variable’s normality [27,28]. The transformation reversed the meaning of the variable such that higher scores indicated poorer functioning. This transformed item was included as an indicator of the physical health latent construct.

##### Body Mass Index (BMI)

BMI is a calculation of an individual’s body weight in kilograms divided by the square of his/her height in meters (kg/m^2^). Although BMI is not a direct measure of body fat, it has been found to be related to health status in older adults e.g., [29]. The WHO provides guidelines for BMI classification categories that are based on health functioning such that <18.50 kg/m^2^ is considered underweight, 18.50–24.99 kg/m^2^ is considered healthy weight, 25.00–29.99 kg/m^2^ is considered overweight and ≥30.00 kg/m^2^ is considered obese [30]. In the current data, BMI was calculated utilizing self-reported height and weight data. BMI was dichotomized to distinguish obese individuals from the rest of the sample as these individuals are at the highest risk for poor health functioning, and individuals with a BMI ≥30.00 kg/m^2^ made up approximately 18.2% of the sample. Individuals with underweight BMI classification (*n* = 62) were excluded from the analyses. These individuals comprised less than 1% of the total sample.

#### 2.2.2. Subjective Well-Being (SWB)

Measures of both cognitive SWB as measured by life satisfaction, and affective well-being as measured by positive and negative affect, were included in the present study.

##### Life Satisfaction

The Satisfaction with Life Scale (SWLS) was used to assess the cognitive component of subjective well-being [31]. Participants are instructed to: “use the scale to indicate the extent to which you agree with each statement. Please be open and honest in your responding.” The questionnaire included five items, such as “in most ways, my life is close to my ideal” that participants rated on a scale from 1—“Strongly agree” to 5—“Strongly disagree”. Response items were reverse scored so that higher scores represented higher levels of life satisfaction. Reliability of the measure from the current data was adequate (Cronbach’s α = 0.87). The five individual items from the scale were included as indicators of a latent construct of life satisfaction, which was one construct in the hierarchical model of SWB.

##### Positive and Negative Affect

The Positive and Negative Affect Schedule (PANAS) was used to assess the affective component of subjective well-being [32]. This measure comprises 10 positive emotion words (e.g., “enthusiastic”), and 10 negative emotion words (e.g., “guilty”), and participants were instructed to rate to what extent they have felt that way over the course of the past few months on a scale from 1—“Very slightly or not at all” to 5—“Extremely”. Reliability from the current data was adequate for positive affect (Cronbach’s α = 0.88) and negative affect (Cronbach’s α = 0.87). To reduce the number of observed variables, the 10 positive affect items were randomly assigned to one of three positive affect parcels and were averaged, and the 10 negative affect items were randomly assigned to one of three negative affect parcels and were averaged. For both positive and negative affect, two parcels included three items, and one included four items. Parceling improves the reliability of a latent construct more so than the use of individual observed items because using individual items has the potential to introduce bias whereas randomly aggregating items into parcels protects against this [8,33]. Thus, the parceling method was used to represent the positive and negative affect latent constructs, which were part of the hierarchical model of SWB.

##### Depression

The short form of the German translation of the Center for Epidemiological Studies—Depression (CES-D) scale was used to assess participants’ level of depressive symptoms [34]. Participants were told: “Below is a list of the ways you might have felt or behaved. Please tell me how often you have felt this way during the past week.” This measure comprised 15 items, such as “I was bothered by things that usually don’t bother me” that participants rated on a scale from 1—“Rarely or none of the time (less than 1 day long)” to 4—“Most or all of the time (5 to 7 days long)”. Higher values indicated higher levels of depressive symptoms. Thus, two positively worded items (i.e., I was in a good mood; I enjoyed life) were recoded. Reliability from the current data was adequate (Cronbach’s α = 0.87). The parceling method was again used to reduce the number of observed variables and parameters to be estimated. Each of the 15 scale items were randomly assigned to one of five parcels and were summed. These five parcels served as indicators of the latent construct of depression.

#### 2.2.3. Engagement in Exercise

Engagement in exercise was assessed through questions about the overall frequency of engagement as well as the number of hours and minutes per week individuals participated in four different exercise categories. Overall frequency of engagement was utilized in the current study to acquire a global sense of exercise engagement and was rated on a scale from 1—“Daily” to 6—“Never” [20]. Responses were reversed so that higher scores represented more frequent engagement. Engagement in these four activities was substantially positively skewed, so a logarithmic transformation of the items was performed, which improved their normality. These four transformed items served as indicators of the latent construct of engagement in exercise. Questions for individual physical activities are included below.

##### Endurance Sports

“How often do you do endurance sports, i.e., swimming, long-distance running, jogging, cycling or similar activities? Please include activities in a fitness studio (aerobics, treadmill, ergometer, etc.).”

##### Team Sports

“How often do you play team sports or games like soccer, volleyball, tennis, handball, basketball, squash, badminton, etc.?”

##### Strength Training/Combat Sports

“How often do you do strength training or combat sports, i.e., weightlifting, bodybuilding, karate, judo, or similar activities? Please include activities in a fitness studio (weights, machines, etc.).”

##### Relaxation/Meditation Exercise

“How often do you do relaxation or meditation exercises, i.e., yoga, autogenic training, progressive muscle relaxation (PMR), tai chi, or qi gong?”

#### 2.2.4. Control Variables

Based on previous research with the present dataset, e.g., [35], age, gender, marital status and socioeconomic status (SES) (as measured by average monthly household income in Euros) were included in the analyses as covariates, where applicable.

### 2.3. Data Analytic Procedure

All data were managed in IBM SPSS Statistics 24.0 [36], and all data were analyzed in Amos 24.0 [37]. Structural equation modeling was used to (1) examine whether age was negatively associated with engagement in exercise, (2) examine whether physical health mediated the relationship between psychological well-being and engagement in exercise, and (3) examine whether age moderated the relationship between physical health, psychological well-being, and engagement in exercise. Goodness of fit for the structural equation models were evaluated using the following criteria: chi-square statistic (χ^2^) in which a small, nonsignificant value indicates better model fit e.g., [8]; however, the chi-square statistic is highly sensitive to sample size, meaning the larger the sample, the more likely it is that chi-square will be significant [38]. Thus, other measures of fit were also evaluated, including the ratio between chi-square and degrees of freedom (χ^2^/*df*), in which values below 5.0 are considered acceptable [39], the comparative fit index (CFI), in which values above 0.95 indicate good model fit [40], and the root mean square error of approximation (RMSEA) in which values below 0.06 [40] or 0.08 [41] are adequate. An alpha level of 0.01 was used for all analyses, and missing data were handled using the full information maximum likelihood estimation method.

## 3. Results

### 3.1. Participant Characteristics

Means and standard deviations of variables of interest, including age and SES, physical health variables, psychological well-being variables, and exercise variables, are included in Table 1. The descriptive information is depicted with columns representing first the total sample and then separated by the specific age groups (i.e., 40–59 years, 60–79 years, 80 years and older). As previously mentioned, the full sample included 50.8% males with the subsamples comprising 47.6% males, 53.3% males and 51.5% males, respectively. Within the full sample, 72.1% of participants were married. Within the 40–59 years, 60–79 years and 80 years and older subsamples, 73.9%, 74.1% and 51.1% of participants were married, respectively. Overall, the average age of the sample was 62.56 (*SD* = 11.92) years with an average monthly income of 2540.02 (*SD* = 2315.56) Euros. Participants had an average of 2.30 (*SD* = 1.83) illnesses, were relatively high functioning with an average score of 83.41 (*SD* = 23.33) on the measure of functional status, and had an average BMI of 26.67 (*SD* = 4.32) kg/m^2^. Table 2 presents the correlations between age and the other variables of interest within the full sample. All correlations between age and the variables of interest were significant and in the expected direction. Most of the variables of interest were significantly correlated with one another.

### 3.2. Preliminary Confirmatory Factor Analyses

Before proceeding with the path and mediation analyses, separate confirmatory factor analyses (CFAs) were conducted with the full sample to ensure that the indicators hypothesized to load onto each latent construct did, in fact, share substantial variance in common. The physical health latent construct was represented by observed variables of self-rated health (β = 0.73, *p* < 0.01), number of illnesses (β = −0.61, *p* < 0.01), functional status (β = −0.81, *p* < 0.01), and BMI (β = −0.21, *p* < 0.01). The hierarchical SWB latent construct was represented by latent constructs of positive affect (β = 0.57, *p* < 0.01), negative affect (β = −0.46, *p* < 0.01), and life satisfaction (β = 0.99, *p* < 0.01). All the variables loading onto each respective lower-order construct were significantly different from zero at the *p* < 0.01 level with standardized loadings ranging from 0.58 to 0.88. The latent construct of depression was represented by five parcels comprising the sum of three randomly assigned items (β_parcel1_ = 0.70, β_parcel2_ = 0.75, β_parcel3_ = 0.82, β_parcel4_ = 0.82, and β_parcel5_ = 0.69, *p*’s < 0.01). The exercise latent construct was represented by observed variables of frequency on several different types of exercise, including endurance sports (β = 0.67, *p* < 0.01), team sports (β = 0.35, *p* < 0.01), strength training/combat sports (β = 0.46, *p* < 0.01), and relaxation/meditation exercises (β = 0.31, *p* < 0.01). The model fit indices for each of the models representing the latent variables of interest are included in Table 3. Each of these models fit reasonably well, with CFIs close to 1 and RMSEAs close to 0. The models fit especially well for the latent constructs of physical health and engagement in exercise, which had nonsignificant chi-square values despite the large sample size. The RMSEA for the latent construct of depression was somewhat high, but considering the satisfactory CFI, the fit was sufficient to proceed with the path analyses using this variable.

### 3.3. Goal #1: Association between Age and Engagement in Exercise

The first goal was to assess whether age was negatively related to frequency of engagement in exercise while controlling for other important sociodemographic variables. We included observed variables for age, gender, marital status and SES in the model as predictors of the latent construct of engagement in exercise. The model fit poorly, and gender and marital status were found to have standardized coefficients close to zero and, thus, were removed from the model. The subsequent model examining whether engagement in exercise was negatively associated with age while controlling for SES was a relatively good-fitting model, χ^2^ = 140.39; *df* = 8; χ^2^/*df* = 17.55; CFI = 0.938; RMSEA = 0.045 (90% CI of RMSEA [0.039, 0.052]). The standardized coefficient of age predicting engagement in exercise was −0.32 (*p* < 0.001). Thus, as age increased, engagement in exercise significantly decreased, even while controlling for the influence of SES, indicating that older adults engaged in exercise to a lesser extent than their younger counterparts.

### 3.4. Goal #2: Physical Health as a Mediator of the Relationship between SWB and Engagement in Exercise

The second goal was to examine whether physical health mediated the relationship between SWB and engagement in exercise (see Table 4). In Model 1, SWB significantly predicted engagement in exercise (β = 0.22, *p* < 0.001) and this model fit the data fairly well, χ^2^ = 1888.35; *df* = 86; χ^2^/*df* = 21.96; CFI = 0.948; RMSEA = 0.051 (90% CI of RMSEA [0.049, 0.053]). In Model 2, after physical health was entered as a mediator, the magnitude of the relationship between SWB and engagement in exercise decreased (from 0.22 to 0.05) and was no longer significant. Model 2 also fit the data well, χ^2^ = 3125.22; *df* = 146; χ^2^/*df* = 21.41; CFI = 0.931; RMSEA = 0.050 (90% CI [0.049, 0.052]). Overall, physical health mediated the relationship between SWB and engagement in exercise. Next, we assessed whether physical health mediated the relationship between depression and engagement in exercise (see Table 4). In Model 3, depression significantly predicted engagement in exercise (β = −0.17, *p* < 0.001) and this model fit the data well, χ^2^ = 744.32; *df* = 26; χ^2^/*df* = 28.63; CFI = 0.965; RMSEA = 0.058 (90% CI of RMSEA [0.055, 0.062]). In Model 4, after physical health was entered as a mediator, the magnitude of the relationship between depression and engagement in exercise decreased in magnitude (from −0.17 to 0.09). Model 4 also fit the data adequately, χ^2^ = 1863.78; *df* = 62; χ^2^/*df* = 30.06; CFI = 0.939; RMSEA = 0.060 (90% CI of RMSEA [0.057, 0.062]). Overall, physical health mediated the relationship between depression and engagement in exercise.

### 3.5. Goal #3: Age as a Moderator of the Mediated Relationship between SWB and Engagement in Exercise

**Figure 1:**
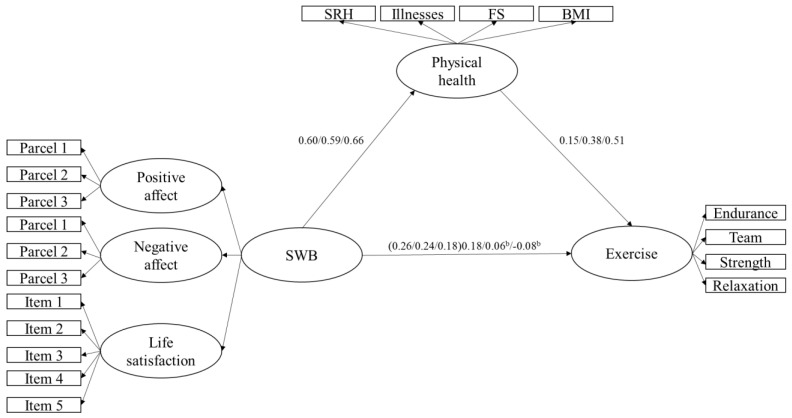
This figure depicts Model 2, in which SWB is a higher-order construct represented by three lower-order latent constructs (i.e., positive affect, negative affect, and life satisfaction). Model 2 represents physical health mediating the relationship between SWB and engagement in exercise across three age groups. The first value before the “/” is the standardized coefficient for the 40–59-year-olds, the middle value between two “/”s is the standardized coefficient for the 60–79-year-olds, and the value after the “/” is the standardized coefficient for the individuals 80 years and older. The values in the parentheses are the standardized coefficients before physical health was entered into the model as a mediator and represents the direct effects between SWB and engagement in exercise. All standardized coefficients are significant at the *p* < 0.01 level unless otherwise specified. Error and unique variance terms have been removed for clarity. ^b^ = *p* > 0.01; SRH = self-rated health; FS = functional status.

**Figure 2:**
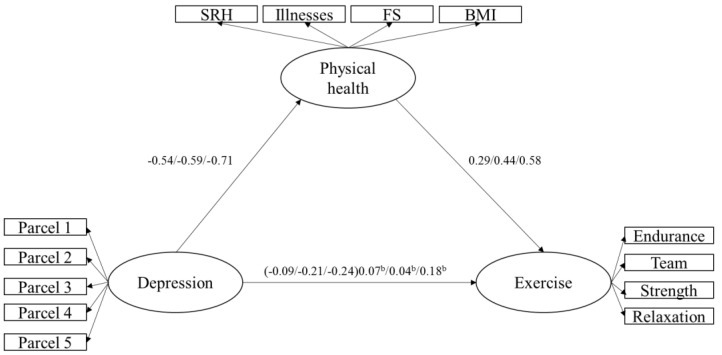
This figure depicts Model 4, a model that represents physical health mediating the relationship between depression and engagement in exercise across three age groups. The first value before the “/” is the standardized coefficient for the 40–59-year-olds, the middle value between two “/”s is the standardized coefficient for the 60–79-year-olds, and the value after the “/” is the standardized coefficient for the individuals 80 years and older. Unless otherwise indicated, all standardized coefficients are significant at the *p* < 0.01 level. Error and unique variance terms have been removed for clarity. ^b^ = *p* > 0.01.

The final goal was to examine whether age moderated the relationships among the variables by examining the differences across three age groups (i.e., 40–59 years, 60–79 years, 80 years and older). Table 5 displays the model fit indices of Models 1, 2, 3 and 4 across the three age groups. Overall, each of these models across the three age groups fit reasonably well, with CFIs close to 1 and RMSEAs close to 0. Figure 1 and Figure 2 show the standardized path coefficients of the relationships between the latent constructs for each age group both before and after physical health was entered as a mediator. As shown in the figures, physical health mediates the relationship between SWB and exercise and depression and exercise. Table 6 presents the standardized path coefficients with 99% confidence intervals to compare the coefficients across age groups. Non-overlapping confidence intervals indicate that the estimates are significantly different from one another at the *p* < 0.01 level. Overall, the influence of physical health as a mediator in the relationship between SWB and engagement in exercise did not differ across age groups. The coefficients of physical health as the mediator were not significantly different from one another across the age groups (as indicated by overlapping confidence intervals). These results are depicted in Figure 1.

Next, we assessed whether age moderated the mediated relationship between depression and engagement in exercise using three age groups (i.e., 40–59 years, 60–79 years, 80 years and older). We found that age did not moderate the mediated relationship because the 99% confidence intervals of the standardized coefficients for each age group overlapped. These findings are depicted in Figure 2.

## 4. Discussion

Overall, the analyses revealed several important findings. First, age was negatively associated with engagement in exercise such that increased age was associated with less engagement in exercise. This finding is consistent with previous research that found similar age-related patterns of exercise [13,42]. This decline in exercise participation across age may be related to changes in health status [13] or a perceived discrepancy between one’s physical capacity and the level of physical ability required for the exercise tasks or to misconceptions about age-related injury risk [43,44]. Additionally, subsequent analyses showed that the relationship between psychological well-being (both SWB and depression) and engagement in exercise was mediated and largely explained by physical health. These findings are consistent with previous research that has noted the particularly strong influence of physical health as a determinant of engagement in exercise [17]. Finally, although age was associated with differences in the magnitude of the mediation, the differences in the coefficients were not significant, indicating that age did not significantly moderate the relationship. Ultimately, these results help to fill a gap noted by previous researchers [10], who indicated that more than one determinant of exercise should be examined at a time. In the current study, we examined both physical health and psychological well-being (measured by SWB and depression) as determinants of exercise and found that physical health played a mediating role in this relationship. We also examined whether this mediation varied across three age groups, which spanned a wide range of ages, including 40 to 93 years. Thus, these findings may have important implications for future exercise research among older adults and interventions.

Foremost, because age was negatively associated with rates of engaging in exercise, there may be some utility to targeting age groups differently in order to encourage exercise. For instance, different approaches and additional resources may need to be developed and allocated for motivating older adults to engage in exercise since they are less likely to be physically active. For example, in some cases, practical barriers prevent older adults from engaging in exercise. There was a paucity of community-level physical activity and exercise programs that target the specific needs of older adults, which may result in older adults’ less frequent engagement in exercise [45]. Thus, the special needs of older adults should be considered when devising strategies of encouraging exercise among older adults. Furthermore, results from the current study highlight the importance of maintaining high levels of physical health from an early age. Since physical health was found to account for the relationship between psychological well-being and engagement in exercise, forming health-promotive habits earlier in life is important since healthy habits perpetuate better health status [15]. Ultimately, these findings emphasize the importance of physical health as a determinant of exercise over and above the influence of psychological well-being.

### 4.1. Limitations

Although this study has observed important patterns in the factors that predict engagement of exercise, there are a few limitations of the research that should be noted. First, the sample consisted of adults residing in Germany, limiting the generalizability of the findings to non-German samples. However, increases in the prevalence in chronic illness and the subsequent negative consequences are occurring in other countries around the world as well. Since exercise is one method of preventing and effectively managing chronic illness, analyses from the current study should be replicated in other countries as well to investigate whether the patterns are comparable. Finally, using only self-report exercise measures without any objective measures of exercise, such as actigraphy data, could be a limitation since self-reported exercise and objective measures of activity are not always strongly correlated e.g., [46]. Future studies may seek to collect objective measures of exercise as well as physical health.

### 4.2. Future Directions

In addition to collecting actigraphy data on physical activity and exercise, future studies may seek to examine explanatory mechanisms between psychological well-being and engaging in more unstructured physical activities, such as physically demanding leisure activities, like gardening, in addition to the more structured exercise activities observed in the present study. Previous research has found that engaging in leisure activities is predictive of positive health and psychological outcomes e.g., [47]. Thus, understanding the specific predictors and explanatory mechanisms predicting engagement in physically demanding leisure activities will be a helpful future direction in this line of research. Future research may also further contribute to this literature by assessing the cross-lagged relationship between physical health, psychological well-being, and engagement in exercise to identify the strength of these predictive relationships over time.

## 5. Conclusions

Overall, the current study contributes important findings to the literature regarding determinants of exercise across the adult life span. Specifically, increased age was related to decreased engagement in exercise. Also, the relationship between psychological well-being (both SWB and depression) and engagement in exercise was mediated and largely explained by physical health. Finally, age did not significantly moderate the mediated relationships, indicating that the relationships were not significantly different across age.

## Figures and Tables

**Table 1:** Means (and Standard Deviations) of Variables of Interest.

Variable	Full Sample *n* = 8136	40–59 Years *n =* 3418	60–79 Years *n =* 4031	80 Years and Older *n =* 687
Age	62.56 (11.92)	50.68 (5.46)	69.20 (5.30)	82.62 (2.48)
Socioeconomic status	2540.02 (2315.56)	2991.11 (2791.57)	2259.77 (1843.37)	1943.15 (1728.43)
Self-rated health	3.50 (0.88)	3.69 (0.85)	3.41 (0.86)	3.08 (0.91)
Number of illnesses	2.30 (1.83)	1.63 (1.49)	2.62 (1.86)	3.60 (1.96)
Functional status ^1^	83.41 (23.22)	91.08 (16.98)	80.74 (23.43)	60.64 (29.83)
Body mass index	26.67 (4.32)	26.32 (4.39)	27.04 (4.31)	26.20 (3.83)
Positive affect	3.52 (0.55)	3.60 (0.54)	3.50 (0.56)	3.31 (0.56)
Negative affect	2.01 (0.55)	2.08 (0.57)	1.97 (0.53)	1.86 (0.52)
Life satisfaction	3.78 (0.75)	3.69 (0.77)	3.83 (0.73)	3.90 (0.70)
Depression	6.45 (6.16)	6.23 (6.17)	6.35 (6.02)	8.13 (6.68)
Endurance sports ^1^	2.95 (1.77)	3.12 (1.69)	2.96 (1.81)	2.03 (1.64)
Team sports ^1^	1.39 (0.88)	1.57 (0.97)	1.30 (0.83)	1.11 (0.50)
Strength training ^1^	1.34 (0.98)	1.45 (1.09)	1.29 (0.94)	1.07 (0.48)
Relaxation exercise ^1^	1.69 (1.34)	1.73 (1.31)	1.68 (1.36)	1.52 (1.31)

Note. ^1^ As mentioned in the Method section, functional status and all of the physical activities were transformed in order to improve their normality for subsequent analyses. However, because the values of the transformed variables are not meaningful as means and standard deviations, the original variables were included in this table to provide a better description of the sample.

**Table 2:** Correlations between Age and Variables of Interest.

Variable	1	2	3	4	5	6	7	8	9	10	11	12
1. Age	1											
2. Self-rated health	−0.24 ^1^	1										
3. Number of illnesses	0.38 ^1^	−0.44 ^1^	1									
4. Functional status	−0.38 ^1^	0.59 ^1^	−0.43 ^1^	1								
5. BMI	0.06 ^1^	−0.18 ^1^	0.16 ^1^	−0.19 ^1^	1							
6. Positive affect	−0.17 ^1^	0.36^1^	−0.22 ^1^	0.30 ^1^	−0.05 ^1^	1						
7. Negative affect	−0.13 ^1^	−0.18 ^1^	0.26 ^1^	−0.11 ^1^	0.00	−0.21 ^1^	1					
8. Life satisfaction	0.11 ^1^	0.33 ^1^	−0.18 ^1^	0.21 ^1^	−0.06 ^1^	0.47 ^1^	−0.39 ^1^	1				
9. Depression	0.07 ^1^	−0.48 ^1^	0.28 ^1^	−0.44 ^1^	0.04 ^1^	−0.38 ^1^	0.40 ^1^	−0.41 ^1^	1			
10. Endurance sports	−0.15 ^1^	0.22 ^1^	−0.09 ^1^	0.24 ^1^	−0.15 ^1^	0.19 ^1^	0.08 ^1^	0.13 ^1^	−0.11 ^1^	1		
11. Team sports	−0.20 ^1^	0.15 ^1^	−0.12 ^1^	0.17 ^1^	−0.07 ^1^	0.13 ^1^	0.01	0.05 ^1^	−0.10 ^1^	0.20 ^1^	1	
12. Strength training	−0.14 ^1^	0.10 ^1^	−0.06 ^1^	0.10 ^1^	−0.06 ^1^	0.11 ^1^	0.05 ^1^	0.02	−0.06 ^1^	0.30 ^1^	0.12 ^1^	1
13. Relaxation exercise	−0.04 ^1^	0.05 ^1^	0.02	0.05 ^1^	−0.12 ^1^	0.14 ^1^	0.07 ^1^	0.06 ^1^	−0.02	0.19 ^1^	0.05 ^1^	0.11 ^1^

Note. ^1^ *p* < 0.01; the transformed variables were not used in this correlational analysis but are included in all structural equation modeling analyses.

**Table 3:** Model Fit Indices for Confirmatory Factor Analyses of Latent Constructs of Interest.

Variable	χ^2^	*df*	*p*	χ^2^/*df*	CFI	RMSEA	90% CI of RMSEA
1. Physical health	2.26	2	0.324	1.13	1.000	0.004	0.000, 0.023
2. Subjective well-being	1128.47	41	<0.005	27.52	0.967	0.057	0.054, 0.060
3. Depression	604.89	5	<0.005	120.98	0.969	0.121	0.113, 0.130
4. Engagement in exercise	5.83	2	0.054	2.92	0.997	0.015	0.000, 0.031

Note. CFI = comparative fit index; RMSEA = root mean square error of approximation.

**Table 4:** Standardized Path Coefficients and *p*-values for Mediation Analyses within the Full Sample.

Model	Standardized Path Coefficient
Model 1:	
SWB → Engagement in exercise	0.22 *
Model 2 ^a^:	
SWB → Engagement in exercise	0.05
Model 3:	
Depression → Engagement in exercise	−0.17 *
Model 4 ^a^:	
Depression → Engagement in exercise	0.09 *

Note. ^a^ = after physical health was entered as a mediator; * = *p* < 0.01; SWB = Subjective well-being.

**Table 5:** Model Fit Indices for Mediation Analyses across Age.

Model	Age Group	χ^2^	*df*	χ^2^/*df*	CFI	RMSEA	90% CI of RMSEA
Model 1: SWB → Engagement in exercise	40–59	594.40	86	6.91	0.966	0.042	0.038, 0.045
	60–79	1009.48	86	11.74	0.947	0.052	0.049, 0.055
	80+	232.51	86	2.70	0.939	0.050	0.042, 0.058
Model 2 ^a^: SWB → Engagement in exercise	40–59	997.86	146	6.84	0.952	0.041	0.039, 0.044
	60–79	1626.46	146	11.14	0.930	0.050	0.048, 0.052
	80+	395.12	146	2.71	0.916	0.050	0.044, 0.056
Model 3: Depression → Engagement in exercise	40–59	227.96	26	8.77	0.978	0.048	0.042, 0.053
	60–79	458.94	26	17.65	0.956	0.064	0.059, 0.070
	80+	102.55	26	3.94	0.954	0.066	0.052, 0.079
Model 4 ^a^: Depression → Engagement in exercise	40–59	635.84	62	10.26	0.953	0.052	0.048, 0.056
	60–79	1041.58	62	16.80	0.931	0.063	0.059, 0.066
	80+	267.27	62	4.31	0.914	0.069	0.061, 0.078

Note. ^a^ = after physical health was entered as a mediator; all χ^2^ values were significant at *p* < 0.01.

**Table 6:** Standardized Path Coefficients (and 99% Confidence Intervals) across Age.

Model	40–59 Years	60–79 Years	80 Years and Older
Model 1:	0.26 *	0.24 *	0.18 *
SWB → Engagement in exercise	(0.18, 0.35)	(0.17, 0.31)	(0.01, 0.34)
Model 2 ^a^:	0.18 *	0.06	−0.08
SWB → Engagement in exercise	(0.07, 0.29)	(−0.04, 0.16)	(−0.36, 0.20)
Model 3:	−0.09 *	−0.21 *	−0.24 *
Depression → Engagement in exercise	(−0.17, −0.007)	(−0.28, −0.14)	(−0.41, −0.06)
Model 4 ^a^:	0.07	0.04	0.18
Depression → Engagement in exercise	(−0.03, 0.16)	(−0.05, 0.13)	(−0.09, 0.44)

Note. * = *p* < 0.01; ^a^ = after physical health was entered as a mediator.
